# Estimates of HIV burden in emergencies

**DOI:** 10.1136/sti.2008.029843

**Published:** 2008-07-22

**Authors:** M Lowicki-Zucca, P B Spiegel, S Kelly, K-L Dehne, N Walker, P D Ghys

**Affiliations:** 1 United Nations Children Fund, New York, USA; 2 United Nations High Commissioner for Refugees, Geneva, Switzerland; 3 Joint United Nations Programme on HIV/AIDS, Geneva, Switzerland; 4 Johns Hopkins Bloomberg School of Public Health, Bethesda, Maryland, USA

## Abstract

**Objective:**

To quantify the proportion of people living with HIV who are being affected by emergencies.

**Methods:**

Emergencies were defined as conflict, natural disaster and/or displacement. Country-specific estimates of populations affected by emergencies were developed based on eight publicly available databases and sources. These estimates were calculated as proportions and then combined with updated country-level HIV estimates for the years 2003, 2005 and 2006 to obtain estimates of the number of men, women and children living with HIV who were also affected by emergencies.

**Results:**

In 2006, 1.8 (range 1.3–2.5) million people living with HIV (PLHIV) were also affected by conflict, disaster or displacement, representing 5.4% (range 4.0–7.6%) of the global number of PLHIV. In the same year, an estimated 930 000 (range 660 000–1.3 million) women and 150 000 (range 110 000–230 000) children under 15 years living with HIV were affected by emergencies. In emergency settings, the estimated numbers of PLHIV in 2003 and 2005 were 2.6 million (range 2.0–3.4 million) and 1.7 million (range 1.4–2.1 million), respectively, representing 7.9% and 5.1% of the global number of PLHIV).

**Conclusions:**

These estimates provide a rationale to ensure that HIV interventions are integrated into rapid assessment of all emergency and preparedness and response plans to prevent HIV infections and address excess suffering, morbidity and mortality among these often overlooked vulnerable groups.

The past decade has seen major improvements in emergency response and huge advances in care and treatment for people living with HIV (PLHIV). However, much less attention has been given towards the overlap between HIV and emergencies due to conflict, natural disasters and displacement (hereafter, generally referred to as “emergencies”).

In 2006, over 40 countries were affected by a humanitarian crisis^i^^1^ with 26 experiencing violent conflicts of high intensity.^2^ By the end of 2006, there were an estimated 14.3 million refugees^ii^ and 24.5 million internally displaced persons (IDPs). In 2007, there were an estimated 33.2 million people living with HIV of whom 2.5 million were newly infected.^3^ Seven of the 15 countries with the largest number of PLHIV in 2005 were affected by major conflict between 2002 and 2006 (table 1); this number increases to 10 countries if the category is broadened to also include humanitarian crises.

**Table 1:** Countries with largest burden of HIV in 2005 and affected by conflict or humanitarian crisis: 2002–6

	PLHIV 2005		Humanitarian crisis	Conflict
	Estimate	Range		
South Africa	5 500 000	4 900 000–6 100 000		
Nigeria	2 900 000	1 700 000–4 200 000		Yes
India*	2 500 000	2 000 000–3 100 000		Yes
Mozambique	1 800 000	1 400 000–2 200 000	Yes	
Zimbabwe	1 700 000	1 100 000–2 200 000	Yes	
United Republic of Tanzania	1 400 000	1 300 000–1 600 000	Yes	
Kenya	1 300 000	1 100 000–1 500 000	Yes	
USA	1 200 000	720 000–2 000 000		
Zambia	1 100 000	1 100 000–1 200 000	Yes	
Democratic Republic of Congo	1 000 000	560 000–1 500 000	Yes	Yes
Uganda	1 000 000	850 000–1 200 000	Yes	Yes
Russian Federation	940 000	560 000–1 600 000	Yes	Yes
Malawi	940 000	480 000–1 400 000	Yes	
Ethiopia	—	420 000–1 300 000	Yes	Yes
Côte d’Ivoire	750 000	470 000–1 000 000		
China	650 000	390 000–1 100 000		Yes

*India estimate of PLHIV is for 2006.^2^

Source: elaboration of data from references 1–3 and 10–15.

How HIV transmission is affected by emergencies is complicated and includes an interconnected mixture of exacerbating and diminishing vulnerability and risk factors that are context specific.^4^^–^7 While the links between conflict and the evolution of HIV prevalence have been studied,^4^^–^6 8 9 another aspect of the overlap of emergencies with the AIDS epidemic—namely, the proportion of people already living with HIV who are affected by emergencies, has not previously been quantified.

In this article, we develop and present regional and global estimates of the number of PLHIV who are affected by emergencies. We then discuss the implications of these estimates for programmatic response and provide recommendations.

## METHODS

Estimates were developed for the years 2003, 2005 and 2006. Five steps were used to derive estimates of HIV-affected populations that were affected by emergencies: (1) Seven emergency-specific databases and sources were used to select countries with emergency-affected populations. (2) For each selected country, the population size affected by the emergency was estimated. (3) Estimates generated in step 2 were transformed into proportions of each country’s overall population. (4) Proportions were applied to existing HIV estimates generated by UNAIDS and the World Health Organization.^3^ (5) Country estimates were aggregated into regional and global estimates and by type of emergency.

The following publicly available databases were used: (1) humanitarian funding appeals; (2) Uppsala conflict database; (3) conflict barometer of the Heidelberg Institute for International Conflict Research (HIIK); (4) Emergency Events Database (EM-DAT); (5) UN High Commissioner for Refugees (UNHCR); (6) the United Nations Relief and Works Agency for Palestine Refugees in the Near East (UNRWA); (7) global internally displaced people (IDP) project database; and (8) World Population Prospects, UN Department of Economic and Social Affairs.

Humanitarian appeals (http://ochaonline.un.org/) include consolidated appeal processes (CAPs) and flash appeals. The CAP is a coordinated programme cycle to analyse context, assess needs and plan prioritised humanitarian responses. A country can be included in a CAP when it is affected by an emergency, be it a manmade or natural disaster. The Uppsala conflict database (http://www.pcr.uu.se/database/index.php) is a free resource of information on armed conflicts in the world. The database includes 121 armed conflicts during the period 1989–2005. The HIIK (http://www.hiik.de/start/index.html.en) continually updates the conflict simulation model (COSIMO) database and the results are published in the annual conflict barometer. Since 1988, the Centre for Research on the Epidemiology of Disasters has been maintaining an EM-DAT (http://www.em-dat.net/) that contains essential data on the occurrence and effects of over 12 800 disasters from 1990 to the present. The database is compiled from various sources, including UN agencies, non-governmental organisations, insurance companies, research institutes and press agencies. UNHCR (http://www.unhcr.org) and UNRWA (http://www.unrwa.org) provide data on refugees. Information provided includes numerical estimates of the number of refugees by host country and country of origin. The database of the Internal Displacement Monitoring Centre (IDMC) (http://www.internal-displacement.org), established in 1998 by the Norwegian Refugee Council (NRC), is the leading international body monitoring conflict-induced internal displacement worldwide. It runs an online database providing comprehensive information and analysis on internal displacement in some 50 countries. Finally, the World Population Prospects, developed by the Population Division of the UN Department of Economic and Social Affairs (http://www.un.org/esa/population/unpop.htm) provides estimates and projections of the total population of each country.

For each reference year, countries with populations affected by disasters, conflict and/or displacement have been identified according to the above-mentioned sources. A country could be featured in more than one of these databases, which reflects that different types of emergencies have affected the population of that country (for example, a country appearing in both the EM-DAT and COSIMO databases would be affected by both natural disasters and conflict). Emergencies generally affect only part of a country. For example, only the north and parts of the east of Uganda have recently been affected by conflict. The same also goes for natural disasters, as for instance with the tsunami in 2004 affecting only parts of India, Sri Lanka, Thailand, Indonesia and Somalia. Estimates of the size of the emergency-affected populations within each country were determined from the available data sources.

The narrative component of humanitarian appeals often contains an estimate of the population in need of humanitarian assistance, although sometimes only the worst-affected population groups are included; when available such an estimate was captured. EM-DAT provides estimates for emergency-affected populations and, thus, the total number of people affected by natural emergencies for each reference year was recorded.

Epidemics and technological accidents were excluded since these types of emergencies would probably have a different impact pattern on HIV-related vulnerabilities from other emergencies such as droughts and floods. UNHCR’s Global Refugee Trends and UNRWA’s annual reports provide information on the number of refugees by country of origin and country of asylum. Similarly, IDMC data provide information on the number of people affected by displacement.

When no population size estimates were available in the above databases, they were generated through web-based searches. Online searches were performed using search engines such as Google, Blackle or Vivisimo for specific keywords, including the type of emergency (for example, conflict, earthquake, drought) and “population” and “affected population” with the reference date. When no estimate was identified through the web-based searches, then the population of the geographical delimitation that most plausibly covered the emergency-affected area (for example, region, province, district or metropolitan area) was taken. Census data or official population estimates by government bodies were searched on the internet and utilised for this purpose. The web-based search was used for only a small number of occasions (that is, Haiti 2005, Russia 2005 and 2006, India 2003, 2005 and 2006).

More than one emergency was sometimes registered for a country in the same year, or more than one estimate for the population affected by one or more emergencies may have been available (for example, the population of the Central African Republic was affected by armed conflict and by floods in 2005, CAP figures for the population affected by drought in Malawi in 2005 and Niger in 2006 differed from those in EM-DAT for the same emergency in the same geographical area and year). In such cases, “low” and “high” scenarios were developed. The “low” scenario was based on the hypothesis that the populations affected by different emergencies overlapped or we used the lowest estimate when more than one source provided a numerical estimate for the affected population. The “high” scenario assumed that populations affected by different emergencies do not overlap and thus, they were summed to provide the total population. A central estimate was developed by calculating the average of the low and high scenarios. For every country, the estimates for the emergency-affected population were expressed as a proportion of the country’s total population, obtained from the World Population Prospects. This resulted in a set of low, central and high proportions.

UNAIDS and WHO provide annual updates on the global HIV epidemic.^3^ Estimates are available for the number of men, women and children <15 years living with HIV. The national estimate for each of these figures of PLHIV, women living with HIV (WLHIV) and children living with HIV (CLHIV) was multiplied by the proportion representing the population affected by an emergency. The UNAIDS plausibility ranges around each country’s HIV estimates were also multiplied by the same proportion.

Since published estimates focus on national populations and do not include refugees, a separate estimate for refugees living with HIV was developed that was also disaggregated for women and children. HIV prevalence estimates for refugees were used when available. Where they were unavailable, the average of the HIV prevalence in the county of asylum and country of origin was used to estimate the refugee prevalence. This is to account for observed tendency of HIV prevalence among refugee populations to near that of host populations in the long run.^5^ When prevalence was so low as to not be reported for the country of origin or asylum, a default figure of 0.05% was used.iii Figures do not change in any meaningful way by using even lower default estimates. For instance, the number of estimated refugees living with HIV obtained by replacing 0.05% with 0.005% changes by less than half a percentage point, not changing in any significant way the regional and global estimates. When data were not available for the country of origin of a given group of refugees, the HIV prevalence among them was calculated as the global average HIV prevalence among all refugees (as estimated in the step before).

Estimates for the number of refugee women living with HIV and for refugee children <15 years living with HIV have never before been generated. Thus, we applied to refugees living with HIV in each region (except North America and Western Europe) the corresponding regional proportions of WLHIV and CLHIV as given by the epidemiological update of UNAIDS,^3^ because the overwhelming majority of refugees living in those regions come from other countries in the same region. Since this is not the case with North America and Western Europe, we applied the global proportions of women and children, to reflect that refugees in those countries come from most regions of the world.

**Figure 1:**
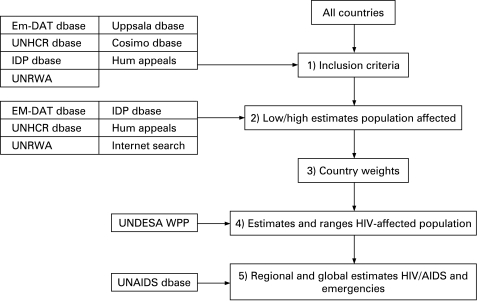
Diagram of the development of the estimates.

A summary of the steps followed by the estimation process is provided in figure 1.

## RESULTS

Table 2 summarises the regional breakdown of emergency-affected people for the three reference years. Globally, almost 350 million people were affected by emergencies in 2003 of which almost two-thirds were in east Asia, particularly because of floods that affected China. The total number of people affected by emergencies in 2005 and 2006 was estimated at 160 million and 185 million, respectively. The region with the numerically most stable population affected by emergencies was sub-Saharan Africa at approximately 50 million affected people for each year, followed by Oceania with approximately 100 000 people.

**Table 2:** Populations affected by conflict, disaster and displacement, central estimate by year

Region	Year		
	2003	2005	2006
Caribbean	240 000	4 500 000	50 000
East Asia	226 900 000	20 100 000	71 100 000
Eastern Europe and Central Asia	4 690 000	2 100 000	1 860 000
Latin America	5 670 000	6 800 000	4 600 000
North Africa and Middle East	9 150 000	7 300 000	5 800 000
North America	800 000	1 300 000	1 000 000
Oceania	100 000	100 000	120 000
South and South-East Asia	49 000 000	69 500 000	45 600 000
Sub-Saharan Africa	50 400 000	54 300 000	53 500 000
Western Europe	2 500 000	1 900 000	1 800 000
Global	349 539 000	167 995 000	185 503 000

The other regions showed significant variations across the three reference years in the absolute numbers of people affected by emergencies, confirming the volatile nature of these events. In percentage terms, besides East Asia, the Caribbean region also showed a very large variation in proportions of people affected by emergencies across the reference years the result primarily of disasters in Cuba and Haiti. In relative terms, however, comparing each region’s annual number of emergency-affected people with the regional average over the three reference years, the regions that manifest the least drastic oscillations around the mean are sub-Saharan Africa and North America.

In 2006, 1.8 (range 1.3–2.5) million PLHIV were affected by conflict, disaster and/or displacement, representing 5.4% (range 4.0–7.6%) of the global total. In the same year, an estimated 930 000 (range 660 000–1.3 million) women and 150 000 (range 110 000–230 000) children under 15 years living with HIV were affected by emergencies. The estimated number of people in emergency settings living with HIV in 2003 and 2005 were 2.6 million (range 2.0–3.4 million) and 1.7 million (range 1.4–2.1 million), respectively, representing 7.9% and 5.1% of the total number of PLHIV.

Still in 2006, 128 000 refugees were estimated to be living with HIV, of whom 69 000 were women and 10 000 were children. Refugees living with HIV accounted for about 7% of the global emergency-affected population living with HIV.

Sub-Saharan Africa accounts for the largest numbers of emergency-affected people living with HIV (between 84.2% and 88.7% through the three reference years), but a relatively smaller proportion of its PLHIV were affected by emergencies than many other regions (10.6%, 6.4% and 7% in 2003, 2005 and 2006, respectively) when this methodology is used. By comparison, in the course of the three reference years, East Asia, Middle East/North Africa and the Caribbean had a higher proportion of their PLHIV who were affected by emergencies (tables 3–5).

**Table 3:** PLHIV affected by emergencies

	2003	Range	% of PLHIV	2005	Range	% of PLHIV	2006	Range	% of PLHIV
Sub-Saharan Africa	2 300 000	1.9 m–3 m	10.6	1 400 000	1.2 m–1.7 m	6.4	1 500 000	1.2 m–2.1 m	7.0
East Asia	92 000	72 000–110 000	16.3	14 000	12 000–16 000	2.0	38 000	30 000–45 000	5.2
Oceania	<1000	<1000–<1000	1.7	<1000	<1000–<1000	1.7	< 1000	<1000–1000	1.4
South and South-East Asia	75 000	26 000–160 000	2.0	120 000	14 000–51 000	3.1	90 000	48 000–160 000	2.3
Eastern Europe and Central Asia	17 000	4600–38 000	1.5	7400	4800–12 000	0.5	6200	<1000–16 000	0.4
Western and Central Europe	15 000	15 000–15 000	2.2	13 000	13 000–14 000	1.9	11 000	11 000–12 000	1.5
Middle East and North Africa	65 000	50 000–84 000	19.9	52 000	39 000–67 000	14.9	48 000	36 000–64 000	13.3
North America	6200	TBD	0.5	7800	TBD	0.6	8200	TBD	0.6
Caribbean	2700	2700–3700	1.3	27 000	27 000–37 000	12.6	<1000	<1000–<1000	0.2
Latin America	18 000	13 000–24 000	1.3	23 000	13 000–40 000	1.5	16 000	8900–28 000	1.0
Global	2 600 000	2 m–3.4 m	7.9	1 700 000	1.4 m–2.1 m	5.1	1 800 000	1.3 m–2.5 m	5.4

TBD, to be determined.

**Table 4:** WLHIV affected by emergencies

	2003	Range	% of WLHIV	2005	Range	% of WLHIV	2006	Range	% of WLHIV
Sub-Saharan Africa	1 200 000	990 000–1.6 m	10.2	770 000	640 000–870 000	6.2	850 000	610 000–1.1 m	6.8
East Asia	22 000	TBD	16.1	3600	1600–TBD	2.1	10 000	TBD	5.5
Oceania	<500	<500–<500	3.5	<500	<500–<500	2.9	<500	<500–<1000	2.5
South and South-East Asia	21 000	5700–36 000	2.1	36 000	14 000–51 000	3.3	28 000	16 000–45 000	2.5
Eastern Europe and Central Asia	3900	1000–9500	1.4	1700	1100–2900	0.5	1500	<500–4100	0.4
Western and Central Europe	6600	TBD	3.6	6000	5900–6100	0.8	6100	6000–6200	3.0
Middle East and North Africa	35 000	26 000–46 000	21.6	28 000	28 000–28 000	16.0	27 000	19 000–36 000	14.6
North America	2600	TBD	0.9	2900	TBD	0.9	4300	TBD	1.4
Caribbean	1100	TBD	1.5	12 000	12 000–12 000	14.3	<500	<500–<500	0.3
Latin America	4500	3400–6700	1.3	7500	7500–7500	2.0	4600	2600–8400	1.2
Global	1 300 000	1 m–1.7 m	8.7	870 000	870 000–870 000	5.7	930 000	660 000–1.3 m	6.1

TBD, to be determined.

**Table 5:** CLHIV affected by emergencies

	2003	Range	% of CLHIV	2005	Range	% of CLHIV	2006	Range	% of CLHIV
Sub-Saharan Africa	230 000	140 000–270 000	10.7	170 000	110 000–170 000	7.9	140 000	100 000–220 000	7.6
East Asia	<500	TBD	11.4	<100	<100–<100	3.3	<500	<500–<500	6.0
Oceania	<100	<100–<100	1.6	<100	<100–<100	2.4	<100	<100–<100	6.9
South and South-East Asia	2500	<500–TBD	2.1	4100	TBD	3.4	2900	1900–4200	2.9
Eastern Europe and Central Asia	<500	<100–<500	1.3	<100	<100–<100	0.5	<100	<100–<500	0.7
Western and central Europe	<1000	<1000–<1000	17.5	<1000	<1000–<1000	19.7	<1000	<1000–<1000	29.9
Middle East and North Africa	5800	2700–7400	22.0	4400	2600–5200	16.5	3300	2600–4600	15.3
North America	<500	<500–<500	3.3	<500	<500–<500	4.1	<1000	<1000–<1000	5.9
Caribbean	<500	<500–<500	1.6	1500	TBD	16.2	<100	TBD	0.3
Latin America	510	<500–<1000	1.3	<1000	<500–1200	2.2	<500	<500–<1000	1.4
Global	240 000	140 000–280 000	11.5	180 000	120 000–180 000	8.7	150 000	110 000–230 000	7.2

TBD, to be determined.

Although the region of Middle East and North Africa has a lower HIV prevalence than Africa and East Asia and a smaller absolute number of PLHIV, it has the highest proportion of PLHIV and WLHAs affected by emergencies.

## DISCUSSION

There were 1.8 million PLHIV in 2006 who were affected by emergencies, representing 5.4% of the global number of PLHIV. Thus, these global and regional estimates show that a significant percentage of PLHIV, including women and children, have been affected by emergencies such as conflict, natural disasters and displacement. In particular, more than 10% of the population living with HIV in four regions—that is, sub-Saharan Africa, Middle East and North Africa, Oceania and the Caribbean, have been affected by emergencies during at least one of the three years studied.

The geographical location of an emergency, as well as the HIV prevalence among the population in that area, can greatly affect the estimates. One hundred thousand people affected by displacement in the Central African Republic with a prevalence of HIV at 6.2%^3^ would impact the estimate to a larger degree than if 10 times the displacement occurred in an area of China where estimated HIV prevalence is less than 0.1%. The 2005 increase in the proportion of PLHIV (including women and children) in the Caribbean is largely because of the political instability that affected Haiti (among the non-African countries with the highest prevalence of HIV in the world) beginning in 2004. Similarly, the decrease in the proportion of PLHIV (including women and children) in East Asia is largely the result of the dramatically reduced number of people who were affected by natural disasters in China in 2005 compared with 2003.

Of all regions, the Middle East and North Africa region has the highest proportion of PLHIV and WLHAs affected by emergencies. This is primarily because of the various emergencies in Sudan, which accounts for 80% of the regional population living with HIV.^iv^

The emergency-affected population size based upon humanitarian appeals and EM-DAT in southern Africa may have underestimated the full extent of southern Africa’s droughts. Since these countries have the highest HIV prevalence levels in the world, the estimates developed in this paper are particularly sensitive to even small changes in the numbers of emergency-affected people in these countries. For instance, in 2003 the Zimbabwe appeal recorded 30 000 people affected by emergencies, while the World Food Programme had already been providing emergency food assistance to more than three million Zimbabweans, about a quarter of the entire population. The utilisation of this latter estimate would have led to a significant increase in the estimated number of people living with HIV affected by emergency in that country.

The highest proportion of emergency-affected CLHIV is estimated in Western and Central Europe, at a significantly higher level than elsewhere. The estimated number of refugee CLHIV is less than 1000. When compared with the very low number of CLHIV overall estimated to live in Western and Central Europe (approximately 3000 children), then the proportion of emergency-affected CLHIV in the region is much higher than that of other regions where HIV infection among children is more prevalent. Even when the comparison is made between Western and Central Europe and North America, it can be observed that the estimated population of CLHIV, the “denominator”, is nearly four times higher (approximately 11 000 children) in the latter, while at the same time the emergency-affected population is lower (approximately 1.8 million and approximately one million in Western and Central Europe and in North America, respectively, in 2006).

Furthermore, in the calculations of HIV prevalence for women and children refugees we applied the same global HIV prevalence to the number of refugees living in Western and Central Europe and in North America because they come from all over the world. However, if the refugee populations in the two regions differed significantly by country or region of origin, then there may be an additional bias (overestimation or underestimation depending upon the HIV prevalence of the countries). Further investigation of this point is needed.

These estimates do not represent trends through the reference years and therefore cannot and should not be interpreted as such. Specifically, one should not conclude that the situation of PLHIV in emergencies has improved between 2003 and 2006. Emergencies are very fluid and often unpredictable phenomena that are subject to radical changes over a very limited time span. For example, natural disasters such as hurricanes, earthquakes and tsunamis can greatly increase the number of PLHIV affected by emergencies from one year to another. This fluidity also applies to conflicts. In north Uganda, the proportion of the population that was displaced by conflict increased from 50% to more than 95% in just a few months in 2002.

Despite the many strengths of this methodology and the resulting estimates, a number of limitations remain.

Humanitarian appeals and the estimates of people affected by emergencies are not sufficiently standardised to make them comparable. The size of the estimated populations in need of humanitarian assistance is often imprecise owing to the difficulties of measuring these populations, especially in non-refugee camp settings. Thus, they could be overestimated or underestimated. Improved methodologies and standards for population size assessments are needed.

An implicit assumption was made that HIV prevalence is uniformly distributed throughout the population in each country. The estimation of HIV prevalence among refugees and, in particular, women and children, use regional as opposed to country averages. This may have contributed to an elevated estimate of the proportion of emergency-affected CLWH in Western Europe. Improved surveillance in refugee settings, especially non-refugee camp situations, will provide more accurate data in the future. The estimation of people affected by emergencies could have been biased upwards according to the possibility of double counting of populations affected by overlapping emergencies or limitations of the data sources.

The methodology presented in this paper nevertheless offers a new tool. Since the level of detail attained surpasses that of estimates in other emergency-related fields, such as child recruitment or education, the latter may be modified accordingly to improve those estimates, as well as to investigate other areas that are currently lacking numerical assessment (for instance, emergency-affected adolescent populations, orphans and others).

This article presents, for the first time, global and regional numbers and percentages of PLHIV, including women and children, who are affected by emergencies. The numbers are sufficiently high to provide a powerful rationale for advocacy towards a more comprehensive inclusion of HIV-related issues by decision makers at the global and regional level, as well as in those countries with recurrent emergencies. Regions and countries must ensure that specific strategies and interventions for HIV among emergency-affected people are integrated into emergency preparedness, rapid assessment and response plans, funding appeals and programmatic responses; to date this has, unfortunately, often not been the case. Programmes to address care of PLHIV, including continuation of antiretroviral services for people already having treatment or mothers and children enrolled in prevention of mother-to-child programmes, should become part of the toolkit of emergency response. The management of HIV-related opportunistic infections must also be an integral part of any response in these contexts.

Increasing programmatic experiences from the field^16^^–^21 provide examples of these approaches and of their results. Since there was a large variability in PLHIVs affected by emergencies owing to the unpredictability of emergencies, preparedness and contingency plans should be undertaken by all countries.

We hope that the quantification of the number and proportion of HIV-affected populations in emergencies will continue to spur discussion and development of relevant programme guidance that addresses the needs and challenges of delivering HIV preventive and curative services in emergency settings.

## Footnotes

i We use the definition of “humanitarian crisis” provided by the School of Peace Culture at the University of Barcelona, which defines it as “… any situation in which there is an exceptional and widespread threat to human life, health or subsistence. Such crises tend to occur in situations of poverty, fragile government and scarcity of food, in which a natural disaster or armed conflict leads to the appearance of a food crisis, disease and the enforced displacement of the population, either within or outside the country, along with an important mobilisation of international resources in the form of aid.” See School of Peace Culture^1^ for more details on the methodology.

ii 9.9 million refugees recorded by UNHCR, plus 4.4 million recorded by UNRWA.

iii In some countries where the HIV prevalence is assumed to be very low, data may be insufficient to provide a country-level estimate. We believed it not to be appropriate to use 0 for this analysis but rather to set a very low default value (in this case 0.05%) as in most countries there are high-risk groups where HIV is present.

iv Sudan is part of the Middle East and northern Africa region according to WHO and UNAIDS.
